# Healthy retirement begins at school: educational differences in the health outcomes of early transitions into retirement

**DOI:** 10.1017/s0144686x19000928

**Published:** 2019-08-05

**Authors:** Kasim Allel, Ana Sofía León, Ursula M. Staudinger, Esteban Calvo

**Affiliations:** 1 Society and Health Research Center and Laboratory on Aging and Social Epidemiology, School of Public Health, Universidad Mayor, Santiago, Chile,; 2 Millennium Nucleus for the Study of the Life Course and Vulnerability (MLIV), Chile,; 3 Department of Economics, School of Business and Economics, Universidad Diego Portales, Santiago, Chile,; 4 Department of Sociomedical Sciences and Robert N. Butler Columbia Aging Center, Mailman School of Public Health, Columbia University, New York, USA; 5 Department of Epidemiology and Robert N. Butler Columbia Aging Center, Mailman School of Public Health, Columbia University, New York, USA

**Keywords:** education, employment, work, retirement, self-reported health, chronic disease, developing countries, lifecourse

## Abstract

The literature on socio-economic variations in the association between retirement timing and health is inconclusive and largely limited to the moderating role of occupation. By selecting the sample case of Mexico where a sizeable number of older adults have no or very little formal education, this study allows the moderating role of education to be tested properly. Drawing on panel data for 2,430 individuals age 50 and over from the Mexican Health and Aging Study (MHAS) and combining propensity score matching models with fixed-effects regressions, this article investigates differences in the health effects of retirement timing between older adults with varying years of education. Subjective health is measured using a self-reported assessment of respondents’ overall health and physical health as a reverse count of doctor-diagnosed chronic diseases. The results indicate that early transitions into retirement are associated with worse health outcomes, but education fully compensates for the detrimental association with subjective and physical health, while adjusting for baseline health, demographics and socio-economic characteristics. In conclusion, formal education during childhood and adolescence is associated with a long-term protective effect on health. It attenuates negative health consequences of early retirement transitions. Policies and programmes promoting healthy and active ageing would benefit from considering the influence of formal education in shaping older adults’ health after the transition into retirement.

## Introduction

Population ageing is frequently perceived as a cause of concern, but various responses are available to address it (Staudinger and Kocka, 2010; Bloom *et al*., 2015; Rowe, 2015). For example, individuals can respond to their longer lives by making greater investments in their human capital through training and prolonged workforce participation, while policy responses include investments in education and promotion of labour-force participation at older ages. A top priority should be to find behavioural and policy responses that simultaneously promote positive economic and health outcomes (Staudinger *et al*., 2016).

Increasing education is perhaps one of the most promising interventions to achieve a combination of productive and healthy ageing (Morrow-Howell *et al*., 2001; Lutz *et al*., 2014). More-educated individuals are better prepared to make use of new technologies and to adjust to changing environments, such as a new job or life after retirement (Jamieson, 2007; Bonsang and Van Soest, 2014; Rehkopf *et al*., 2017; Narushima *et al*., 2018). Formal education is defined in this article as total years of formal education completed starting from elementary school.

The non-pecuniary returns to education are explained in the economic literature through the ‘allocative efficiency model’, which refers to situations in which more-educated individuals choose a more efficient mix of inputs (*e.g*. time and money) to maximise health (or any given outcome) relative to less-educated individuals facing similar circumstances (Grossman, 2006; Oreopoulos and Salvanes, 2011). Numerous studies in high-income countries have used exogenous variation in accessibility to formal education as a natural experiment, finding that good health is a key non-pecuniary benefit from additional schooling (Lleras-Muney, 2005; Currie and Moretti, 2007; Cutler and Lleras-Muney, 2008). A smaller set of studies has identified the mechanisms underlying the link between formal education and health outcomes (Ross and Mirowsky, 1999; Cutler and Lleras-Muney, 2010; Vogl, 2014). This literature, however, is not specific to the health effects of retirement timing. The present article adds to the literature on non-pecuniary returns on formal education by highlighting that further support for the allocative efficiency model may lie in testing how years of education affect the association between retirement timing and health status after retirement.

In particular, the present study examined how formal education moderates (or differentiates) the association between retirement timing and health in a country that hosts a full (and not a restricted) range of educational exposures; in this case, Mexico. By focusing on Mexico, we remedy data constraints in the extant literature focusing on high-income countries which are characterised by a restricted range of years of education. Extant literature is not only limited to high-income countries, but also fragmented, with one group of studies focusing on the relationship between education and health (Mäki *et al*., 2014) and another group of studies concentrating on the health effects of retirement (Fisher *et al*., 2016); the latter often ignoring the socio-economic differences in the association between retirement and health or only considering the moderating role of occupation, not education (Iparraguirre, 2014). As literature directly testing the moderation effect of education on the association between retirement timing and health is virtually missing, we reviewed literature on the lifecourse health effects of education, health effects of retirement timing and specific studies on occupational variations in the health effects of retirement timing to inform the hypotheses of this study.

In the following sections, we review these literatures, formulate research hypotheses and justify the selection of Mexico as our case study. Next, we discuss our methodological approach. After presenting our results, we discuss policy and practical implications for ‘societies of longer lives’ (Staudinger, 2015). Most notably, we emphasise how policies concerned with healthy and positive ageing are inextricably linked to salient antecedents such as the timing of retirement, as well as to earlier antecedents such as formal education.

## Literature review

### Lifecourse health effects of education

Numerous studies document that education is strongly associated with health over the lifecourse (Luo and Waite, 2005; Jamieson, 2007; Cutler and Lleras-Muney, 2008, 2010; Mäki *et al*., 2014). Education is strongly associated with psychological, social and financial resources that are helpful to deal with health shocks (Kawachi *et al*., 2010). Consistently, numerous studies have documented that more formal education has preventive effects as it lowers health risk, improves cognition and prolongs life expectancy, regardless of being in the labour force or not (Luo and Waite, 2005; Lutz and Samir, 2011; Majer *et al*., 2011; Mäki *et al*., 2014; Cutler *et al*., 2015). Not only formal education, but also lifelong learning through volunteering and training seem to support mental health and cognitive development among adults and older adults (Ball *et al*., 2002; Baltes *et al*., 2006). However, there is also evidence that the protective health effects of education may weaken with age (Van Dijk *et al*., 2008).

### Health effects of retirement timing

Regarding the health effects of retirement timing, a large fraction of this literature suggests transitions that happen too early to have detrimental effects on health, which are partly explained by losses in financial, social and psychological resources (Siegrist *et al*., 2007; Dhaval *et al*., 2008; Zins *et al*., 2011; Behncke, 2012; Kuhntopf and Tivig, 2012; Van der Heide *et al*., 2013; Van der Noordt *et al*., 2014; Fisher *et al*., 2016). Although it is clear that this relationship goes both ways, studies using panel data and an instrumental variable approach suggest that retirement timing causes changes in physical and mental health (Bonsang and Klein, 2012; Bonsang *et al*., 2012; Calvo *et al*., 2013; Kajitani *et al*., 2016; Mazzonna and Peracchi, 2016). However, the benefits of working beyond normative ages are not clear (Calvo *et al*., 2013). Usually, education is controlled in such analyses, and is not conceptualised as possibly moderating the health effect of retirement timing (Fisher *et al*., 2016).

### Socio-economic variation in the association between retirement timing and health

The lack of empirical evidence about the moderating effect of education on the health outcomes of retirement timing may partly be explained by the fact that studies are largely restricted to high-income countries, where ‘no’ or ‘little education’ is such a rare event that the moderating role of education cannot be properly tested. Thus, studies that inform the association between retirement timing and health typically control for education, but they do not investigate education as a potential moderator of the health effects of retirement timing and often focus just on retirement status instead of the timing of retirement (Fisher *et al*., 2016).

For example, a recent study by Hessel (2016) in 12 European countries analysed the effects of retirement on self-rated health, limitations in activities of daily living and the presence of chronic conditions, combining an instrumental variable approach with models stratified by gender and educational level. The role of education as a moderator, however, was inconclusive. On the one hand, retirement was associated with significantly worse self-reported health among highly as compared to low-educated women (but not men), suggesting that *less* education had a protective effect for women. On the other hand, education neither modified the significant association between retirement and increases in functional limitations (for both genders) nor did it modify the lack of association between retirement and the presence of chronic conditions. In another study by Majer *et al.* (2011), education was not directly modelled as a moderator of the health effects of retirement timing either, but the results suggested that individuals with higher levels of education lived in better health before retirement and also lived longer afterwards compared to those with lower educational levels. These observed socio-economic inequalities in health favour the higher educated, but it is unclear whether or not they may moderate the health consequences of retirement timing.

Studies examining socio-economic variations in the health effects of retirement have paid less attention to education and instead focused on the type and prestige of the occupation from which individuals retire as a potential moderator of health effects of retirement (Iparraguirre, 2014). The study samples are similarly restricted to high-income countries and the findings are also inconclusive. A fairly large group of studies provides evidence that individuals who retire from jobs with lower *prestige* and worse working conditions are in worse health after retirement than those who retire from jobs with higher prestige and better working conditions (Mein *et al*., 2003; Nooyens *et al*., 2005; Neuman, 2006; Chung *et al*., 2009). In the United States of America (USA), a study using an instrumental variable approach concluded that retirement had no strong association with health, though may slightly increase the likelihood of depression for men retiring from a low-skill blue-collar occupation (Neuman, 2006). Another study in the USA documented that retirement from a physically demanding job (which typically indicates lower SES) was associated with a decrease in physical activity, while retiring from a sedentary job was associated with an increase in physical activity, and that these associations were further exacerbated by wealth, which is another indicator of socio-economic status (SES) (Chung *et al*., 2009). Similarly, a study in the Netherlands found that retirement was significantly associated with increases in weight and waist circumference among individuals retiring from physically active jobs, but not among those retiring from sedentary jobs (Nooyens *et al*., 2005). Among British civil servants, retirement at age 60 was not significantly associated with physical health, and if anything was weakly associated with mental health improvements among higher-grade occupations (Mein *et al*., 2003). Finally, retiring from jobs involving more complex tasks, which are typically available to individuals with more education, seems to be associated with better cognitive outcomes in the USA (Fisher *et al*., 2014), Japan (Kajitani *et al*., 2016) and many European countries (Mazzonna and Peracchi, 2016).

A smaller group of studies that differentiate the health effects of retirement by occupation reached mixed results. Relative to British civil servants in low occupational grades, those in high grades experienced better mental health but poorer physical health trajectories after retirement (Chandola *et al*., 2007). In France, harmful health behaviours such as heavy alcohol consumption increased around retirement and decreased in the years thereafter, but these changes varied in complex ways by type of occupation across males and females (Zins *et al*., 2011). Males in manual low-SES occupations remained more stable in heavy alcohol consumption before and after retirement and females in managerial high-SES and clerical low-SES occupations (not in intermediate-SES occupations) did not experience a decrease after retirement.

Yet, another study in the USA using an instrumental variable approach found no clear-cut association between retirement and health or cognition for white-collar workers and, if anything, a beneficial effect of retirement for blue-collar workers (Coe *et al*., 2012). To our knowledge, this is the only study that suggests that higher occupational status may make the health effects of retirement worse.

In sum, whether education differentiates the association between retirement timing and health remains unclear (Scharn *et al*., 2018). The lack of empirical evidence on the moderating role of education can be partly attributed to the fact that studies are largely restricted to high-income countries, where few older adults have little or no education. Studies considering the impact of type and prestige of occupation on the association between retirement and health are also restricted to high-income countries (Iparraguirre, 2014). Higher SES, as measured by the type and prestige of occupation, seems to be weakly associated, if anything, with better health outcomes of retiring (Mein *et al*., 2003; Nooyens *et al*., 2005; Neuman, 2006; Chung *et al*., 2009), but the results are inconclusive (Chandola *et al*., 2007; Zins *et al*., 2011; Coe *et al*., 2012). These weak and inconclusive results are consistent with the fact that socio-economic differences in health based on occupation seem to decrease after retirement more than those based on education (Marmot and Shipley, 1996).

### Health outcome specificity

Previous literature calls for studies to focus on specific health outcomes, as the association between retirement timing and health, as well as the pathways connecting them, are outcome-specific (Iparraguirre, 2014; Fisher *et al*., 2016). Relative to physical health outcomes, previous evidence suggests that subjective health outcomes are fairly sensitive to SES-related exposures, including educational level (Maas *et al*., 2006; Hessel, 2016), cognitive inequalities (Borgonovi and Pokropek, 2016), financial resources and material disadvantage (Franks *et al*., 2003), as well as social connectedness and resilience (Stewart *et al*., 2019). Physical health outcomes are also sensitive to these and other SES-related exposures, but they ‘get under the skin’ albeit through more complex and longer pathways (Ferraro and Shippee, 2009; Miller *et al*., 2009; Hertzman and Boyce, 2010; Phelan *et al*., 2010; Matthews and Gallo, 2011). Therefore, considering specific outcomes and potential differences between them is important to a more comprehensive and nuanced understanding of the socio-economic variation in the association between retirement timing and health.

## Research hypotheses

Overall, the literature reviewed suggests that it is plausible to hypothesise that more education protects individuals and buffers the negative health consequences of early transitions into retirement over and above the effect of the type of occupation. We further hypothesised that the moderating effect of education is stronger for subjective than physical health consequences of early transitions into retirement.

## Mexico as a case study

We suggest that part of the dearth of studies and inconsistencies in the extant literature may have to do with the limited educational variance observed in high-income countries and consequently selected a country that displayed a broader range of educational levels from none to college degrees and beyond. We assumed that having a wider range of educational levels available would represent a fairer test of the possible compensatory effect of education, that is, more years of formal education would buffer the negative health consequences of early transitions into retirement. Specifically, we selected Mexico as a case study to explore the role of education in explaining differences in the subjective and physical health effects of retirement timing.

Compared to most high-income countries, Mexico is ageing at a faster rate while its older population has lower levels of schooling and health (Palloni *et al*., 2002; Wong and Palloni, 2009; United Nations, 2015). Thus, Mexico allows us to include a sizeable group of individuals with low and very low levels of formal education in the analysis (in our sample, 24.03% of older adults working in the formal economy have no education). This percentage cannot be found in most high-income countries, where virtually all older adults have completed high school and a substantial fraction had access to tertiary education. In this challenging scenario, Mexicans typically retire in their early sixties, with only half of them having contributed to social security and many experiencing poor health outcomes (Aguila *et al*., 2011; Murill and Venegas, 2011).

## Methods

### Data collection

**Figure 1.**
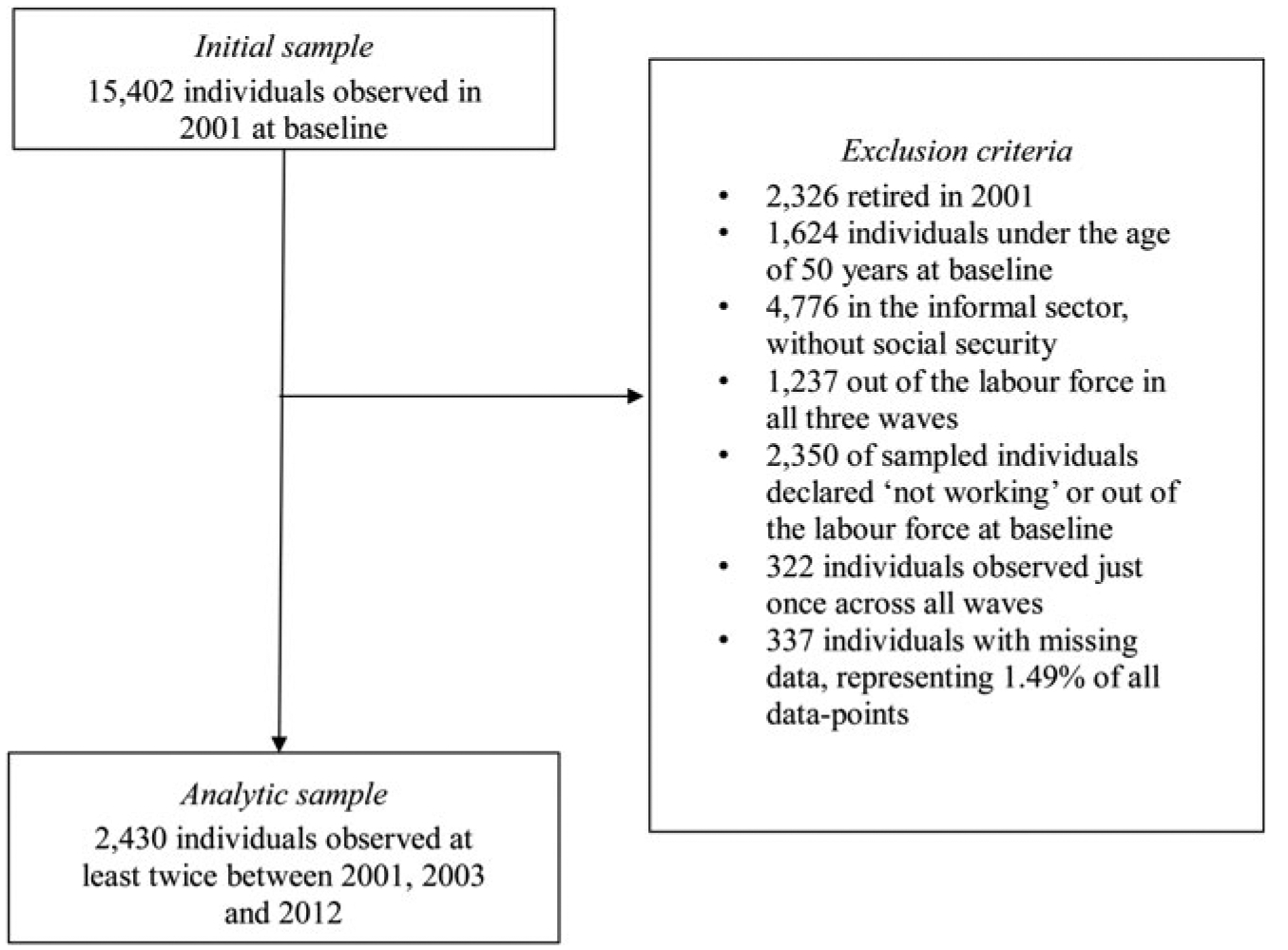
Sample construction.

We used nationally representative panel data of older adults age 50 and over and their spouses from the Mexican Health and Aging Study (MHAS). With support from the National Institute of Health, MHAS followed up 21,421 individuals in 2001, 2003 and 2013 (Instituto Nacional de Estadística y Geografía, 2012; Wong *et al*., 2015). We restricted our sample to 2,430 individuals aged 50 and over surveyed in 2001 (baseline year of the study), participating in the formal labour force and social security system at baseline, and neither retired nor dead during the observation period (*see* Figure 1). Selectivity effects by socio-demographics and health variables were overall fairly small (*see* Figure A1 in the online supplementary material). If anything, it is worth noting that relative to excluded individuals, those in the analytic sample have higher SES as measured by education but lower SES as measured by occupation, and are more likely to have employed spouses.

### Measurements of health outcomes

We used subjective and physical health as main dependent variables. Subjective health was measured using a five-point Likert scale ranging from ‘poor’ to ‘excellent’. Our physical health index is a reverse count of the following chronic conditions: high blood pressure or hypertension, diabetes or high blood sugar, chronic lung disease except asthma, cancer or malignant tumour except skin cancer, heart problems, strokes, and arthritis or rheumatism. This variable ranges from zero to seven, with a higher value indicating better health or absence of chronic diseases.

### Measurements of formal education and retirement timing

To measure formal education, we used years of education completed. To measure retirement timing we used retirement status, current age, current age squared, and interaction terms between retirement status and each of the age variables. Retirement status was computed as a dichotomy indicating that the respondent was receiving retirement benefits or not working because of retirement. Current age was measured in years, centred at 60 and divided by 10 for analytical purposes.

### Covariates

We included a wide range of socio-economic, functioning and lifestyle measures as time-varying covariates in the fixed-effects models. Income was calculated as the sum of annual earnings from business, real estate, capital goods, salary, transfers and pensions, measured in $1,000 units of 2010 Consumer Price Index-adjusted US dollars, and logarithmically transformed to improve normality and linearity. Spousal employment status was measured with dummy variables indicating a non-employed spouse, an employed spouse and no spouse as the reference category. Being out of the labour force status was measured as a dichotomy indicating spells out of the labour force for reasons other than retirement. Functional limitations were measured on a 0–5 scale by counting any difficulties in performing the following activities of daily living: dressing, going to the bathroom, eating, going to bed and walking several blocks. Psychological health was based on a reverse count of eight depressive symptoms from a reduced version of the Center for Epidemiological Studies Depression Scale (CES-D), including positive and negative affects, as well as somatic symptoms. Smoking status was measured as a categorical variable indicating individuals that currently smoke, ever smoked and never smoked as the reference category. Alcohol behaviour was measured as a categorical variable too, using abstainers as the reference category and indicating three types of drinkers: light (<1 drink per day), moderate (1–2.5 drinks per day) and heavy (>2.5 drinks per day). The inclusion of a wide range of covariates was intended to minimise selectivity and omit variable biases.

In the propensity score estimation we included all the covariates mentioned above, combined with gender, ethnicity and type of occupation, all measured as time-invariant covariates at baseline. Gender was measured as a dichotomy indicating women. Ethnicity was also a dichotomy indicating whether or not the respondent speaks an indigenous dialect. Life-time occupation type was measured at baseline for the main job and classified in three groups: blue-collar (agriculture/forestry/fishing, mechanics/repairing, construction industry, extractor, army operators and members of the army), pink-collar (sales occupations/office workers/support and administrative services such as cleaning, protection, food preparation, health services and personal services) and white-collar occupations (professional occupations and directors) as the reference category. Because the goal of the propensity score estimation is an accurate prediction and not a parsimonious explanation, we used all variables regardless of statistical significance.

### Statistical analyses

In order to analyse how having more or less years of formal education moderated the later-life health outcomes of early transitions into retirement, we estimated separated longitudinal linear regression models for subjective and physical health, employing propensity score matching to account for treatment selectivity. For each individual *i* at time *t*, the health effects of retirement timing were estimated through the model formalised in Equation (1):

(1) $$Y_{it}=\alpha_{0}+\beta_{1}\times {\text{Retirement}}_{it}+\beta_{2}\times {\text{Age}}_{it}+\beta_{3}\times {\text{Retirement}}_{it}\times {\text{Age}}_{it}+\beta_{4}\times {\text{Age}}_{it}^{2}+\beta_{5}\times {\text{Retirement}}_{it}\times {\text{Age}}_{it}^{2}+\beta_{6}\times {\text{Retirement}}_{it}\times {\text{Education}}_{it}+\beta_{7}\times {\text{Retirement}}_{it}\times {\text{Education}}_{it}\times {\text{Age}}_{it}+\beta_{8}\times {\text{Retirement}}_{it}\times {\text{Education}}_{it}\times {\text{Age}}_{it}^{2}+\beta_{9}\times {\text{Education}}_{it}\times {\text{Age}}_{it}+\beta_{10}\times {\text{Education}}_{it}\times {\text{Age}}_{it}^{2}+X_{i}+u_{i}+e_{it}$$

where *Y*_*it*_ is the predicted health of respondent *i* at time *t*, *α*_0_ is the constant term or average health, *β* represents the estimated effects for the corresponding variable, Retirement_*it*_ is a variable indicating the status of being retired, Age_*it*_ is measured in years centred to the mean, Education_*it*_ indicates years of education of the respondent, *X*_*i*_ is a vector containing all time-invariant covariates, and *e*_*it*_ and *u*_*i*_ represent time-varying and time-invariant error terms, respectively. The three-way interaction terms between retirement status, years of education, and both linear and quadratic age $\left(\beta_{7}\times {\text{Retirement}}_{it}\times {\text{Education}}_{it}\times {\text{Age}}_{it}\;\;\;\text{and}\;\;\;\beta_{8}\times {\text{Retirement}}_{it}\times {\text{Education}}_{it}\times {\text{Age}}_{it}^{2}\right)$ are needed to test whether formal education buffers the detrimental health outcomes of retirement timing. Performing these estimations separately for subjective and physical health outcomes, and (also separately) plotting predicted health values for individuals that retired and remained in the labour force and their differences in predicted health values by age, allowed exploration of whether or not the moderating effect of education is stronger for subjective than physical health consequences of early transitions into retirement.

Because health crises are one of the possible reasons forcing people out of their jobs at earlier ages, the health effects of retirement timing can be overestimated unless we addressed the endogeneity of retirement timing to health. We addressed this issue by estimating fixed-effects regression models using propensity score weights, without changing the weights across waves (Imai and Kim, 2019). Propensity score techniques weight each individual from the analytic sample using a score corresponding to the estimated probability of receiving the treatment (in this case retiring over the observed period), conditional on individuals’ characteristics at baseline, such that the weighted distribution of the observable characteristics is the same for the treated and untreated groups (Rosenbaum and Rubin, 1983). By weighting individuals using their conditional probability of retirement given a comprehensive set of characteristics observed at baseline year 2001, we adjusted our estimation of the average treatment effect on the treated for individual differences at baseline that could be related to retirement.

**Figure 2.**
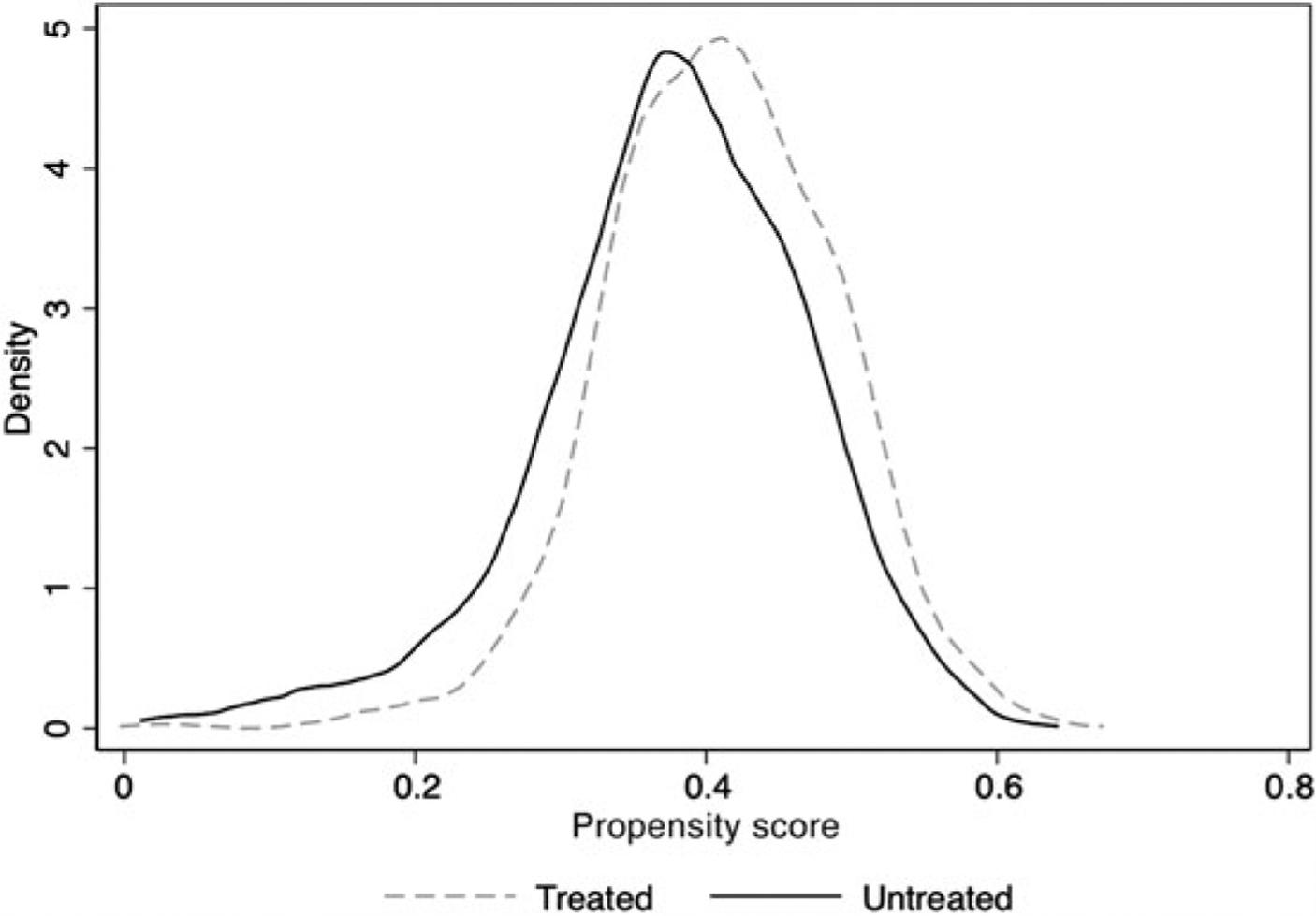
Kernel distribution of the propensity score estimation. *Notes*: Propensity score estimation includes the following independent variables: subjective health and its squared term, physical health and its squared term, functional health limitations and its squared term, years of formal education, current age in years centred with the corresponding squared terms, income logged, spouse working status, having no spouse, sex, indigenous ethnicity, and blue-collar and clerical occupations.

Specifically, for each individual we estimated the conditional probability of being retired during the observed period (*i.e*. receiving treatment) through the logistic model formalised in Equation (2): ln

(2) $$\text{ln}\left[\frac{e\left(z_{i}\right)}{1-e\left(z_{i}\right)}\right]\;=\;\text{ln}\left[\frac{\text{Pr}\left({\text{Retired}}_{i}=1|Z_{i}\right)}{1-\text{Pr}\left({\text{Retired}}_{i}=1|Z_{i}\right)}\right]=\alpha_{0}+\beta_{k}Z_{i}$$

where e(*z*_*i*_) is the propensity score based on the probability of being retired (receiving treatment), conditional on a series of individual characteristics at baseline included in vector *Z*_*i*_. *Z*_*i*_ includes health measures, and functioning, lifestyle, demographic and socio-economic characteristics measured at baseline. For all continuous variables we also included squared terms to obtain the most accurate propensity score in case of non-linear effects on the probability of being retired. The resulting propensity scores have similar distributions for treated and untreated individuals, as indicated by the kernel distribution of the propensity score estimation in Figure 2, suggesting that propensity score estimates will yield appropriate and fully sample-weighted results (Dehejia and Wahba, 2002).

After obtaining the propensity score (PS), we calculated weights for each individual using Equations (3) and (4):

(3) $$\text{Treated case weight}\;:\;\frac{1}{\text{PS}}$$

(4) $$\text{Untreated case weight}\;:\;\frac{1}{1-\text{PS}}$$

For each of our outcomes of interest, subjective and physical health, we estimated fixed-effects versions of the model shown in Equation (1) using Weighted Least Squares (WLS; Rubin, 1997; Hirano and Imbens, 2001; Freedman and Berk, 2008) and adjusting standard errors for clustering within the same household (Kaufman and Rousseeuw, 2009). We present conservative fixed-effects model for physical and subjective health (Hausman test *p* < 0.001). Because physical health is a count variable and is somewhat left-skewed (*see* Figure A2 in the online supplementary material), we compared our WLS results to those obtained using weighted Poisson regression, finding similar results for the coefficients of interest (*see* Table A2 in the online supplementary material). We also estimated random-effects models adding time-invariant covariates; these models reached similar results for the coefficients of interest, but smaller confidence intervals for the figures given their more parsimonious nature (results available from the authors upon request).

## Results

Table 1 shows the descriptive statistics for the first wave, last wave and average across all three 2001, 2003 and 2012 waves. These descriptive statistics suggest that - as to be expected for this age range - both health outcomes significantly decrease between the first and last waves. Also, they show that, on average, respondents have slightly more than six years of education, and that 51.03 per cent retired over the observed period of 11 years. Covariates behave as expected, but it is worth noting the increasingly high proportions of non-employed spouses and blue-collar workers, as well as the high number of functional limitations.

Results from the propensity score models are presented in Table A1 in the online supplementary material. They suggest that higher subjective health, education and income, as well as ever or current smoking status, significantly increased the probability of being retired, while being single and higher age showed the opposite association. Overall, the predicted probability of retiring ranged from 0.01 to 0.60 (mean = 0.38, standard deviation = 0.06).

Table 2 summarises the core results of this study based on fixed-effects regression models that incorporate the predicted probabilities of retirement as weights. The constant suggests that individuals who are working, have an employed spouse, never smoked or drank, have no formal education, and have mean values in all continuous variables, have an average subjective health of 1.99 (on a scale ranging from 0 to 4) and an average physical health of 5.99 (on a scale ranging from 0 to 7). The main effects of retirement timing suggest that better health outcomes are associated with not being retired, and that younger age is associated with better physical health.

However, there are significant interactions between the status of being retired, current age and education (joint tests of significance *p* < 0.001). The two-way interaction between retirement and age suggests that earlier retirement is associated with worse health outcomes. However, the three-way interactions including education suggest that the associations of earlier retirement with worse health outcomes are stronger for individuals with lower levels of education. Notably, this finding is above and beyond the differences in reasons for early retirement.

**Figure 3.**
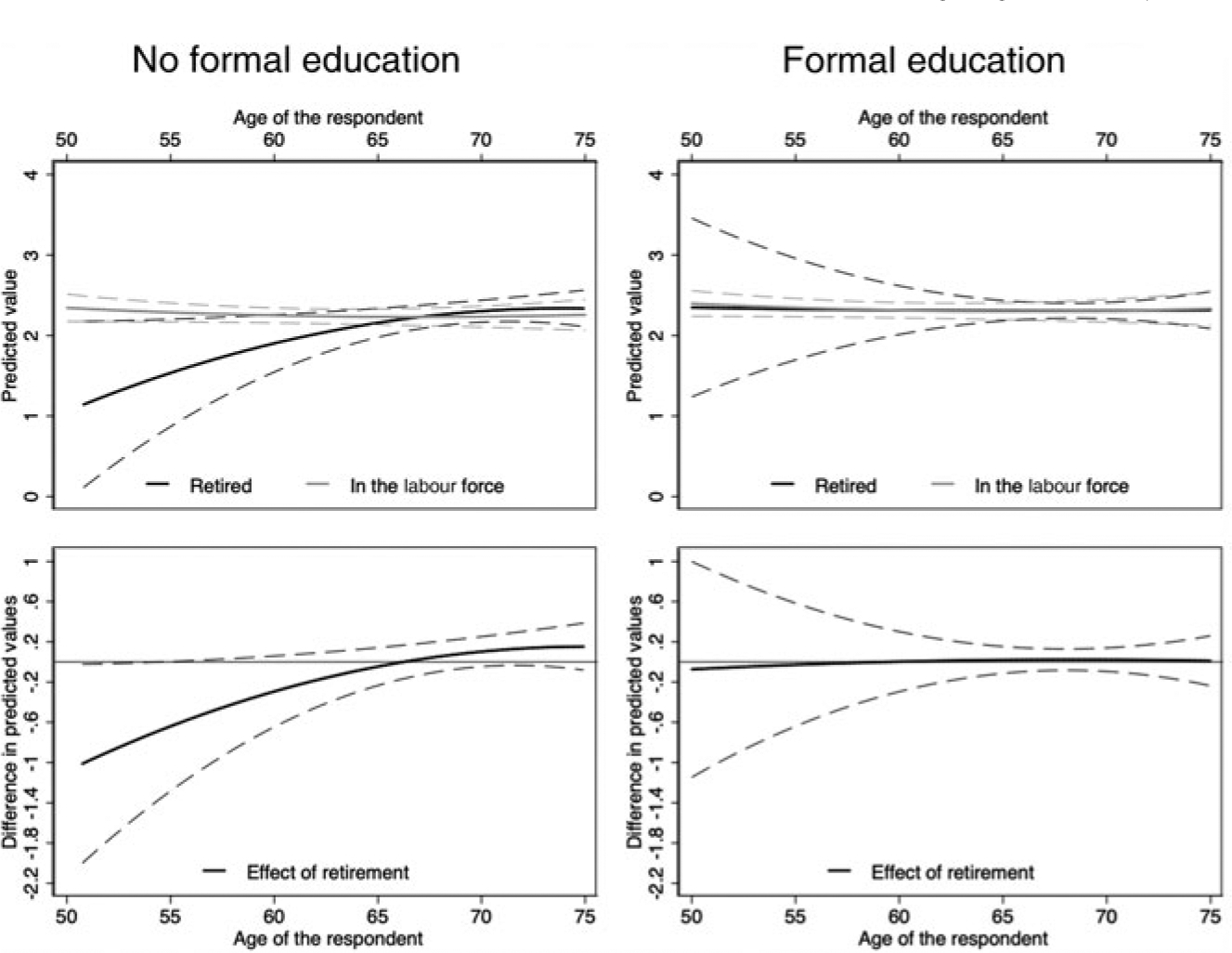
Subjective health effects of retirement timing for individuals with and without formal education. *Note*: Dashed lines show the 95 per cent confidence intervals.

**Figure 4.**
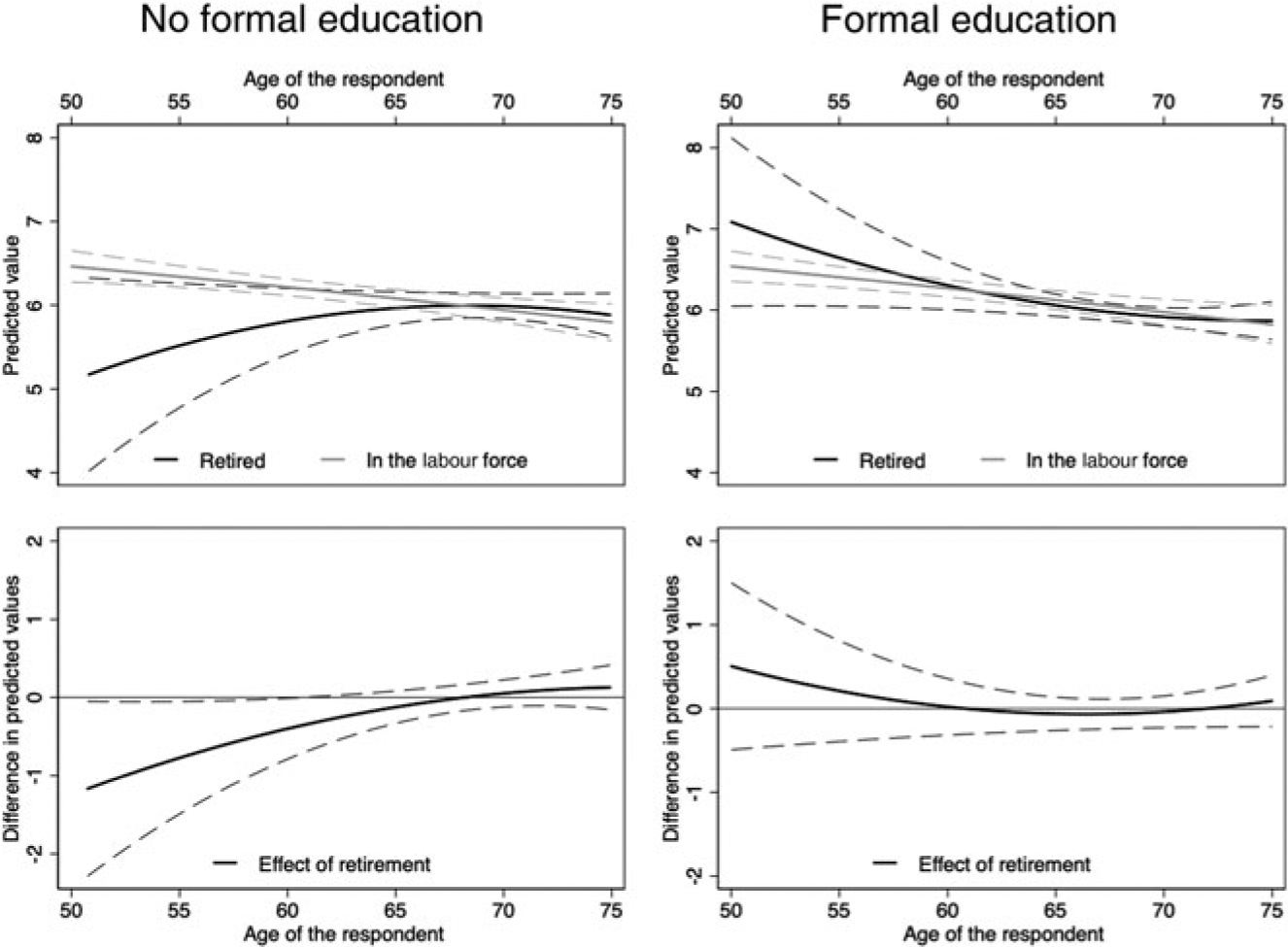
Physical health effects of retirement timing for individuals with and without formal education. *Note*: Dashed lines show the 95 per cent confidence intervals.

Figures 3 and 4 illustrate these results by plotting predicted health values by age for individuals that retired and those that remained in the labour force (top panels) and the difference between the two in predicted health values (bottom panels). The difference in predicted values between those retired and those still in the labour force can be interpreted as the health effect of retiring *versus* remaining in the labour force at a specific age. Dashed lines show the 95 per cent confidence intervals for these estimations. These results are presented for individuals with and without formal education (right and left panels, respectively).

Figure 3 shows that having any formal education fully compensates for the detrimental association between earlier retirement and *subjective health*. The left panel shows that retiring too early is detrimental for the subjective health of individuals without formal education, but at age 66.26 individuals that retired and those that remained in the labour force have the same point estimate in subjective health, suggesting that continued employment into older ages has no significant association with subjective health. The right panel suggests that retirement timing is not significantly associated with the subjective health of individuals with formal education.

Figure 4 displays analogous information for *physical health*. Similarly, a retirement that happens too early is associated with worse health among individuals with no education, but at age 68.1 individuals that retired and those that remained in the labour force have the same point estimate in health, suggesting that continued employment into older ages has no significant association with physical health (left panel). Also consistent with results on subjective health, there is no association between retirement timing and physical health among individuals with formal education (right panel).

Overall, these results are consistent with previous literature suggesting that formal education is associated with better late-life health, as well as supporting our hypotheses that formal education buffers the negative (subjective and physical) health consequences of early transitions into retirement. The hypothesis that the moderating effect of education is stronger for subjective than physical health was inconclusive, as it was only supported when using random-effects models, but not when using fixed-effects models.

## Discussion

This study tested the protective effect of formal education on the health consequences of early transitions into retirement. In order to assess the full power of education, we used a representative sample of older adults in Mexico, a middle-income country that is characterised by a wider variation in years of education in the population than in most high-income countries. We found, as others before, that there was a beneficial effect of formal education on later-life health (Lleras-Muney, 2005; Luo and Waite, 2005; Majer *et al*., 2011; Mäki *et al*., 2014; Vogl, 2014). Due to the wide spread of years of education in our sample, however, we were for the first time able to find a strong buffering effect of formal education on this association between early transitions into retirement and worse health. This moderating role of education on the association between retirement and health is stronger and more consistent than what has been previously observed in one study for education (Hessel, 2016) and in multiple studies for occupation as a marker of SES (Mein *et al*., 2003; Nooyens *et al*., 2005; Neuman, 2006; Chandola *et al*., 2007; Chung *et al*., 2009; Zins *et al*., 2011; Coe *et al*., 2012). The latter finding is consistent with the fact that socio-economic differences in health based on occupation seem to decrease after retirement more than socio-economic differences based on education (Marmot and Shipley, 1996).

We did not find strong evidence suggesting that the moderating effect of education varied by health outcome. Prior evidence documented that subjective outcomes are more sensitive to socio-economic exposures (Franks *et al*., 2003; Maas *et al*., 2006; Stewart *et al*., 2019; Borgonovi and Pokropek, 2016; Hessel, 2016), while physical health outcomes involve more complex and longer pathways for SES-related exposures to ‘get under the skin’ (Ferraro and Shippee, 2009; Miller *et al*., 2009; Hertzman and Boyce, 2010; Phelan *et al*., 2010; Matthews and Gallo, 2011). Based on our results we conclude, however, that the association between early transition into retirement and worse health is fully buffered by education for both subjective and physical health.

Potential mechanisms that may explain the moderating effect of formal education include the differentials between higher- and lower-educated individuals in terms of cognitive and non-cognitive skills, lifestyles, financial and informational resources, quantity and quality of activities, social connections and environmental exposures (Cutler and Lleras-Muney, 2010; Stenholm *et al*., 2014; Van Rijn *et al*., 2014; Vogl, 2014; Fisher *et al*., 2016; Staudinger *et al*., 2016). In exploratory follow-up analyses (available upon request), we found that this protective effect of education stems partly from the higher concentration of resources and the nature of post-retirement activities performed by individuals with more formal education relative to those with less formal or without education. Individuals with formal education accumulate wealth and resources (Kawachi *et al*., 2010), allowing them to cope better with the transition into retirement. Consistent with previous literature, we found that by controlling for financial resources, individuals with more education seem to allocate their available resources in a more efficient way in terms of the production or protection of their health by engaging in a greater variety and higher quality of post-retirement activities (Grossman, 2006; Oreopoulos and Salvanes, 2011). These results are novel in highlighting a previously overlooked dimension of the non-pecuniary benefits of formal education by demonstrating that basic formal education during childhood and adolescence is associated with better health roughly half a century later, when they are older adults and transitioning into retirement. More research is needed, however, to understand the causal pathways connecting retirement timing and health, and the mechanisms that explain the moderating effect of formal education.

These findings have policy and practical implications for low- and middle-income countries facing a rapidly ageing population without universal schooling. Mexico and other countries where schooling is not universal, such as China and India (United Nations, 2013), would benefit from considering formal education as a potential long-term intervention to improve the health of older adults and protect them from potential negative effects of transitions into retirement that happen too early. Our results highlight that policies and interventions promoting healthy ageing should consider formal education as an integral antecedent of later-life health and also a potential tool to address socio-economic disparities in health among lower- and middle-income countries (Borgonovi and Pokropek, 2016). They also suggest that individuals with little or no formal education need more support during their transition into retirement in order to experience similar health outcomes as individuals with formal education.

Despite these important findings, this research has some limitations that we would like to acknowledge. The main limitations are that both health outcomes are self-reported and that at the time of the analyses there were only three waves of data available with an unusually uneven time lag between them. However, conducting this study in a middle-income country like Mexico, where numerous older adults have little or no formal education, allowed us to explore the protective effect of the most basic educational intervention; that is, formal education. Future research could explore differences in other health-related outcomes and the effects of retirement timing by other policy-relevant variables, such as job quality, partial retirement, pension adequacy and lifelong learning. Generating work environments that reinforce health, promoting partial retirements, improving the availability and generosity of old-age pensions, and promoting lifelong learning might constitute additional opportunities to improve health for older adults and to facilitate healthier transitions into retirement (Calvo, 2016; Staudinger *et al*., 2016; Azar *et al*., 2018; Calvo *et al*., 2018).

## Conclusion

Extant literature documents that education is strongly associated with health throughout the lifecourse (*e.g*. Jamieson, 2007; Lutz and Samir, 2011; Majer *et al*., 2011; Vogl, 2014; Cutler *et al*., 2015) and that early transitions into retirement have average detrimental effects on health (*e.g*. Bonsang and Klein, 2012; Calvo *et al*., 2013; Fisher *et al*., 2016; Mazzonna and Peracchi, 2016). This study contributes to existing literature by documenting that formal education continues to be associated with health during the transition to retirement, and more specifically that formal education offsets the detrimental association between early transitions into retirement and both subjective and physical health. This novel evidence suggests that part of the non-pecuniary returns to interventions promoting formal education and literacy campaigns include improving health outcomes later in life, especially during the transition from work to retirement, a critical period for the health trajectories of older adults.

## Supplementary Material

Supplementary materials

## Figures and Tables

**Table 1.** Descriptive statistics

	First wave		Last wave		All waves	
Variables	Mean	SD	Mean	SD	Mean	SD
Health outcomes:						
Subjective health	2.50	0.87	2.36	0.87	2.41	0.86
Physical health	6.23	0.89	5.84	1.04	6.13	0.94
High blood pressure/hypertension (%)	34.26		49.09		38.27	
Diabetes/high blood sugar (%)	14.42		28.37		19.20	
Cancer or malignant tumour (%)	1.76		5.14		2.55	
Chronic lung disease (%)	5.89		7.56		5.67	
Heart problems (%)	2.27		6.11		3.12	
Stroke (%)	1.48		3.26		1.76	
Arthritis/rheumatism (%)	16.37		15.74		15.40	
Psychological health	5.44	2.33	5.32	2.55	5.33	2.43
Formal education:						
Years of education	6.51	4.78	6.50	4.71	6.46	4.75
Retirement timing:						
Retirement status (%)	–	–	51.03	0.50	14.86	0.36
Current age	57.90	6.45	68.27	5.69	61.54	7.66
Covariates:						
Income (in US $1,000, not logged)	11.28	83.32	7.24	50.26	9.53	64.98
Employed spouse (%)	46.29		14.31		30.94	
No spouse (%)	22.89		31.58		23.01	
Spouse not employed (%)	30.82		54.11		46.05	
Not in the labour force (%)	–	–	27.60	0.45	19.42	0.40
Functional limitations index	2.91	1.23	3.14	1.31	2.97	1.25
Psychological health	5.43	2.33	5.31	2.55	5.33	2.43
Never smoked (%)	54.13	0.49	61.87	0.49	57.39	0.49
Ever smoked (%)	26.82	0.44	27.51	0.45	26.21	0.44
Current smoker (%)	19.05	0.39	12.47	0.33	16.40	0.37
Abstainer (%)	61.10	0.49	77.34	0.42	69.33	0.46
Light drinking behaviour (%)	31.13	0.46	18.03	0.38	24.07	0.43
Moderate drinking behaviour (%)	4.44	0.21	3.49	0.18	4.07	0.20
Heavy drinking behaviour (%)	3.32	0.18	1.14	0.11	2.54	0.16
Women (%)	53.57		55.86		54.15	
Indigenous ethnicity (%)	4.82		5.13		4.85	
Professional worker (%)	16.50		16.29		16.44	
Clerical/sale/service worker (%)	34.10		33.9		34.88	
Blue-collar worker (%)	49.40		49.81		48.68	
N	2,430		1,834		6,276	

*Notes*: Raw values before transformations are reported for all variables, unless explicitly noted. Percentages are reported for all dichotomous variables. SD: standard deviation.

**Table 2.** Panel regression results for the average effects on health

	Subjective health			Physical health		
Variables	Coeff.	SD	*p*	Coeff.	SD	*p*
Retirement timing:						
Retirement status	−0.39	0.18	0.030	−0.45	0.19	0.016
Age	−0.06	0.06	0.337	−0.26	0.06	0.000
Age squared	0.04	0.03	0.205	−0.01	0.04	0.779
Interactions:						
Retirement × Age	0.59	0.29	0.039	0.62	0.33	0.060
Retirement × Age squared	−0.20	0.11	0.059	−0.19	0.13	0.133
Retirement × Years of education	0.04	0.02	0.050	0.05	0.02	0.009
Retirement × Years of education × Age	−0.06	0.04	0.146	−0.10	0.04	0.014
Retirement × Years of education × Age squared	0.02	0.02	0.454	0.04	0.02	0.018
Age × Years of education	0.00	0.01	0.947	−0.01	0.01	0.355
Age squared × Years of education	0.00	0.01	0.896	0.00	0.01	0.991
Covariates:						
Income (in US $1,000, logged)	0.12	0.07	0.064	0.07	0.07	0.292
No spouse	0.04	0.07	0.579	−0.26	0.07	0.000
Spouse not employed	−0.06	0.03	0.045	0.05	0.03	0.087
Not in the labour force	−0.06	0.03	0.068	−0.05	0.04	0.143
Functional limitations index	−0.04	0.01	0.000	−0.01	0.01	0.385
Psychological health	0.07	0.01	0.000	0.05	0.01	0.000
Ever smoked	0.00	0.03	0.889	0.04	0.04	0.265
Current smoker	0.08	0.05	0.096	0.22	0.06	0.000
Light drinking behaviour	0.09	0.03	0.004	0.04	0.03	0.219
Moderate drinking behaviour	−0.05	0.07	0.446	0.10	0.06	0.106
Heavy drinking behaviour	0.17	0.08	0.034	0.19	0.09	0.043
Constant	1.99	0.07	0.000	5.99	0.08	0.000

*Notes*: N (individuals) = 2,430; N (observations) = 6,276. Reference categories for categorical variables are employed spouse and professional/managerial worker. Continuous variables are mean-centred. Coeff.: coefficient. SD: standard deviation.
