# Supplementary material for: Healthy retirement begins at school: educational differences in the health outcomes of early transitions into retirement
Source: Ageing Soc. Author manuscript; Available in PMC 2021 Jun 9. (PMC8188825; doi:10.1017/s0144686x19000928)

Running Head: EDUCATION, RETIREMENT, AND HEALTH

**Healthy Retirement Begins at School:**

**Educational Differences in the Health Outcomes of Early Transitions into Retirement**

**APPENDIX**

*Table A1. Propensity score logistic estimation of the probability of retirement*

| Variables | Coeff. | SD | p-value |
| --- | --- | --- | --- |
| *Health outcomes* |  |  |  |
| Subjective health | 0.48 | 0.21 | 0.021 |
| Subjective health squared | -0.07 | 0.04 | 0.053 |
| Physical health | 0.17 | 0.43 | 0.696 |
| Physical health squared | -0.02 | 0.04 | 0.544 |
| *Formal education* |  |  |  |
| Years of education | 0.02 | 0.01 | 0.024 |
| *Covariates* |  |  |  |
| Current age (centred) | -0.03 | 0.07 | 0.722 |
| Current age squared (centred) | -0.33 | 0.08 | 0.000 |
| Income (in $1,000) | 0.01 | 0.00 | 0.024 |
| Income Squared (in $1,000) | 0.00 | 0.00 | 0.054 |
| No spouse | -0.37 | 0.10 | 0.000 |
| Spouse not employed | -0.16 | 0.09 | 0.091 |
| Functional limitations index | -0.11 | 0.28 | 0.690 |
| Functional limitations index squared | 0.01 | 0.04 | 0.772 |
| Psychological health | 0.10 | 0.07 | 0.150 |
| Psychological health squared | -0.01 | 0.01 | 0.079 |
| Ever smoked | 0.24 | 0.09 | 0.012 |
| Current smoker | 0.28 | 0.11 | 0.009 |
| Soft drinking behaviour | 0.10 | 0.09 | 0.248 |
| Moderate drinking behaviour | -0.04 | 0.19 | 0.844 |
| Heavy drinking behaviour | 0.38 | 0.22 | 0.081 |
| Women | 0.04 | 0.08 | 0.610 |
| Indigenous ethnicity | 0.08 | 0.18 | 0.660 |
| Blue-collar worker | 0.07 | 0.12 | 0.551 |
| Clerical/sale/service worker | -0.15 | 0.14 | 0.286 |
| *Constant* | -1.19 | 1.31 | 0.362 |
| *Number of observations* | 2,430 | | |

*Notes*: Reference categories for categorical variables are employed spouse and professional/managerial worker.

*Table A2. Poisson regression results for the average effects on physical health*

| Variables | IRR | SD | p-value |
| --- | --- | --- | --- |
| *Retirement timing* |  |  |  |
| Retirement status | 0.93 | 0.01 | 0.000 |
| Age | 0.96 | 0.01 | 0.000 |
| Age squared | 1.00 | 0.00 | 0.601 |
| *Interactions* |  |  |  |
| Retirement x Age | 1.11 | 0.02 | 0.000 |
| Retirement x Age squared | 0.97 | 0.01 | 0.000 |
| Retirement x Years of education | 1.01 | 0.00 | 0.000 |
| Retirement x Years of education x Age | 0.98 | 0.00 | 0.000 |
| Retirement x Yrs. of educ. x Age squared | 1.01 | 0.00 | 0.000 |
| Age x Years of education | 1.00 | 0.00 | 0.178 |
| Age squared x Years of education | 1.00 | 0.00 | 0.956 |
| *Covariates* |  |  |  |
| Income (in $1,000 and logged) | 0.99 | 0.01 | 0.028 |
| No spouse | 0.96 | 0.01 | 0.000 |
| Spouse not employed | 1.01 | 0.00 | 0.001 |
| Not in the labour force | 0.99 | 0.00 | 0.003 |
| Functional limitations index | 1.00 | 0.00 | 0.174 |
| Psychological health | 1.01 | 0.00 | 0.000 |
| Ever smoked | 1.01 | 0.00 | 0.025 |
| Current smoker | 1.04 | 0.00 | 0.000 |
| Soft drinking behaviour | 1.01 | 0.00 | 0.016 |
| Moderate drinking behaviour | 1.02 | 0.01 | 0.001 |
| Heavy drinking behaviour | 1.03 | 0.01 | 0.000 |
| *Constant* | - | - | - |
| *Number of individuals* | 2,430 | | |
| *Number of observations* | 6,276 | | |

*Notes*: Poisson model estimated using inverse-probability-weighting and robust standard errors. IRR stands for incidence rate ratios.

*
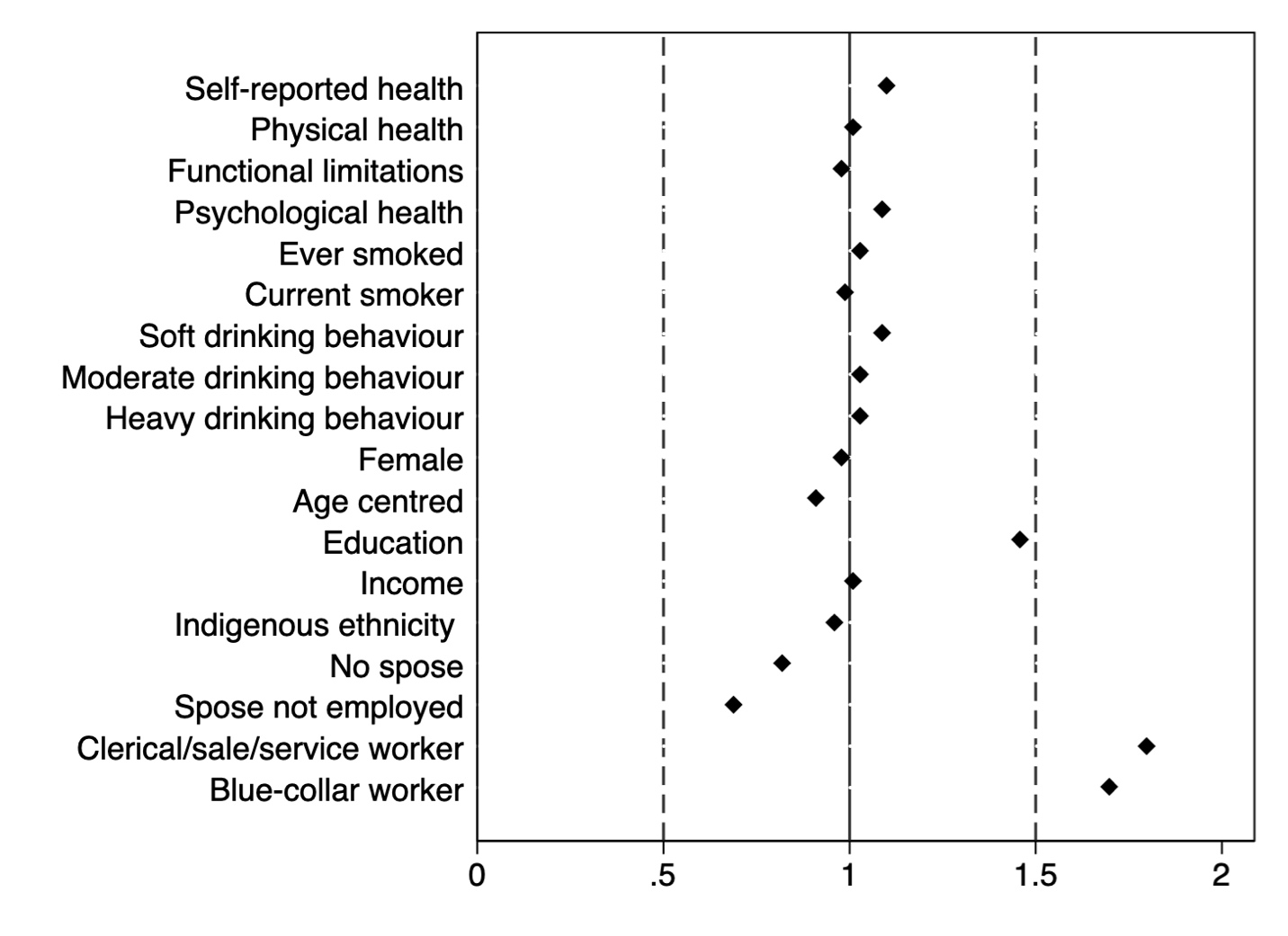
*

*Figure A1: Logistic regression results for sample selectivity at baseline (N= 13,541)*

*Notes:* Standardized odds ratios reported for individuals in our analytic sample (n=2,430) relative to MHAS individuals that were not included in the sample (n=11,111). Reference categories are White collar worker and employed spouse.

**
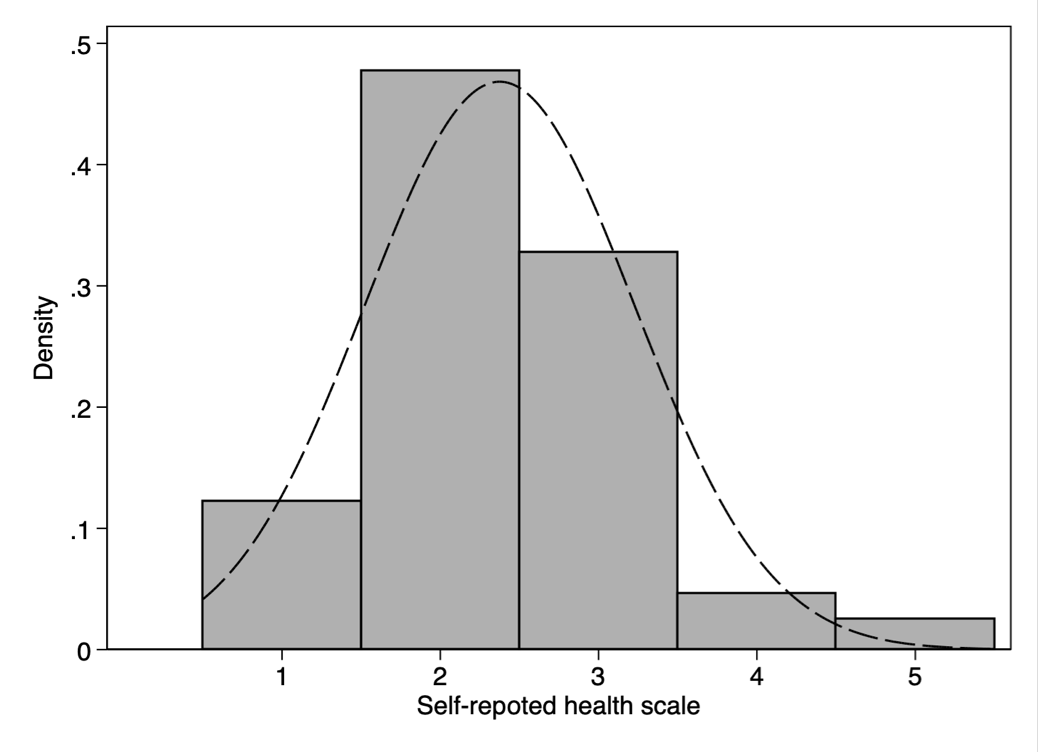
**

**
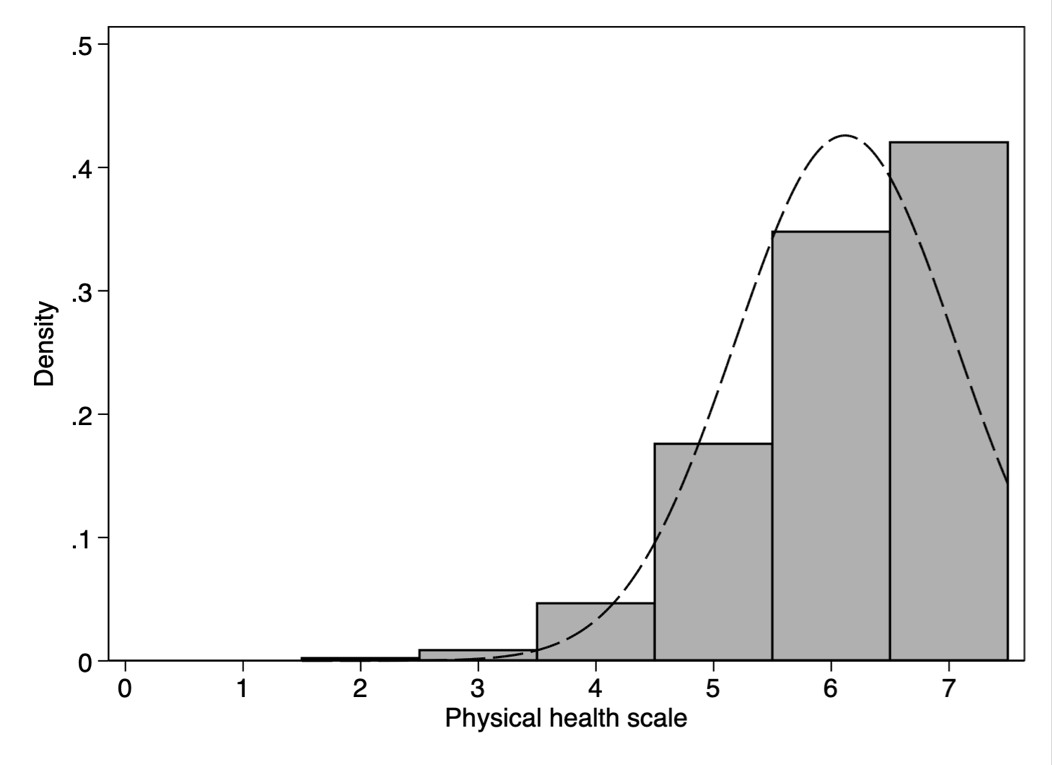
**

*Figure A2. Distributions of subjective and physical health*

***Output for comment 1b by reviewer 1***


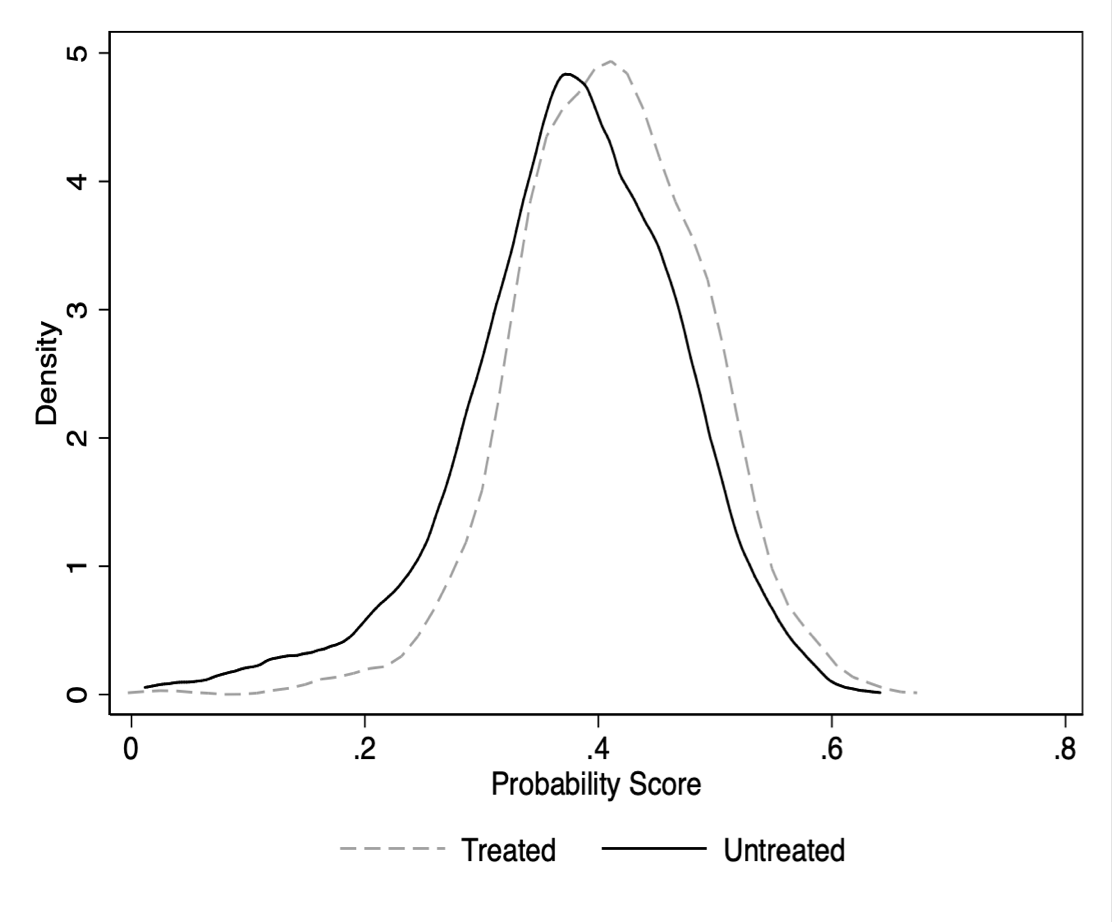


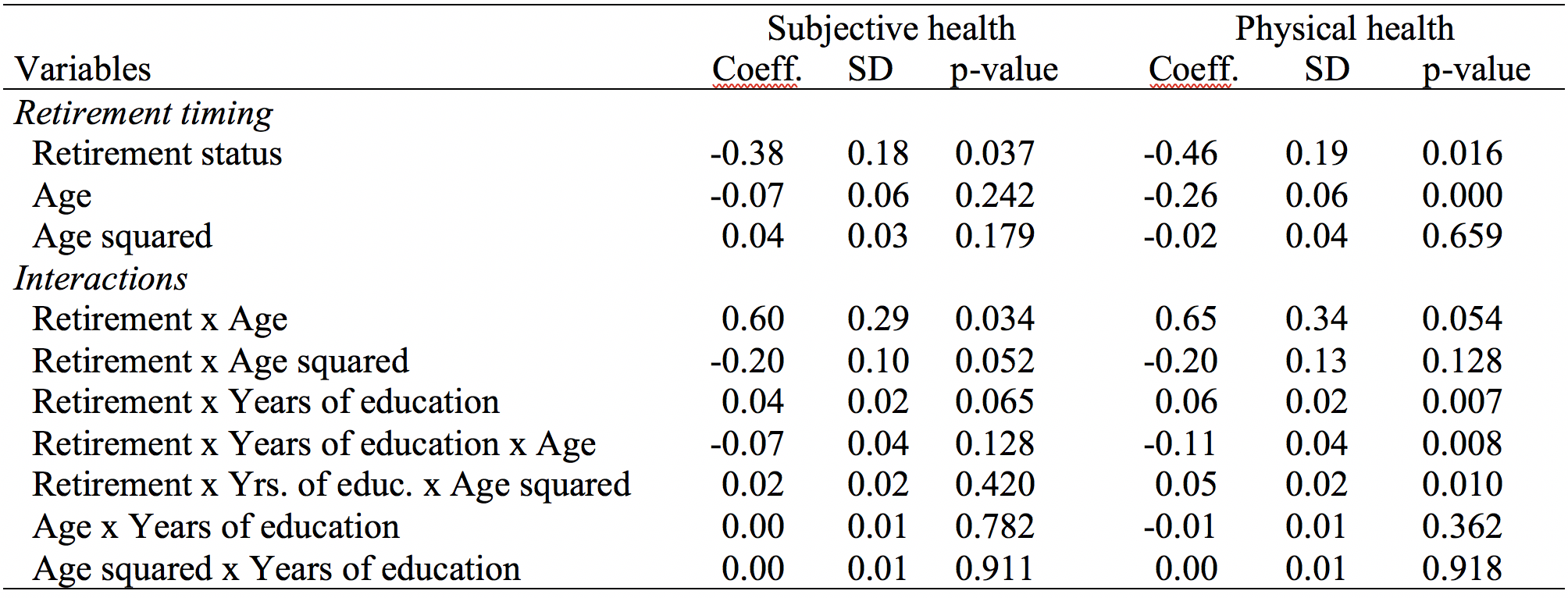

Supplement: Supplementary materials [file NIHMS1572371-supplement-Supplementary_materials.docx]
